# Pharmacokinetics of Doxycycline in Alpacas After Intravenous and Subcutaneous Administration

**DOI:** 10.3390/antibiotics14030247

**Published:** 2025-03-01

**Authors:** José Martínez, Pedro Marín, David A. Egas, Juan Llivi-Marcatoma, José Miguel Mira-Naranjo, Elena Badillo, María Teresa Yuste, Elisa Escudero, Juan Sebastián Galecio

**Affiliations:** 1 Department of Pharmacology, Campus de Espinardo, University of Murcia, 30100 Murcia, Spain; jose.martinez10@um.es (J.M.); pmarin@um.es (P.M.); mariateresa.yuste1@um.es (M.T.Y.); 2 Colegio de Ciencias e Ingenieras, Universidad San Francisco de Quito, Cumbayá 170901, Ecuador; degas@usfq.edu.ec; 3 Escuela de Medicina Veterinaria, Facultad de Ciencias Pecuarias, Escuela Superior Politécnica de Chimborazo, Panamericana Sur Km 1 1/2, Riobamba 060106, Ecuador; juan.llivi@espoch.edu.ec (J.L.-M.); josem.mira@espoch.edu.ec (J.M.M.-N.); 4 Escuela de Medicina Veterinaria, Colegio de Ciencias de la Salud, Universidad San Francisco de Quito, Cumbayá 170901, Ecuador; jgalecio@usfq.edu.ec

**Keywords:** alpacas, bioavailability, doxycycline, pharmacokinetics, subcutaneous, tetracyclines

## Abstract

**Background/Objectives**: Doxycycline, a tetracycline-class antibiotic, is commonly used across various species to treat infections caused by susceptible bacteria. However, pharmacokinetic data on its use in alpacas remains limited. This study aimed to investigate the pharmacokinetics of doxycycline following intravenous (IV) and subcutaneous (SC) administration in alpacas. **Methods**: A randomized crossover study (n = 6) was employed, with dosages of 5 mg/kg and 20 mg/kg after intravenous and subcutaneous administration, respectively. Blood samples were collected at predetermined times up to 96 h after both routes of administration. Plasma doxycycline concentrations were determined using validated high-performance liquid chromatography with a UV detector and then analyzed based on non-compartmental pharmacokinetic methods. **Results**: All alpacas maintained optimal health and general condition throughout the trial period. After intravenous administration, the V_z_ value (0.90 L/kg) indicated a good distribution of this antibiotic in the alpacas. The maximum concentration value (C_max_) after SC administration of doxycycline was 1.40 µg/mL, reached at 1.92 h. Low bioavailability (F = 36.83%) of doxycycline was observed after SC administration. **Conclusions**: PK/PD ratios calculated from the pharmacokinetic data obtained, at a dose of 20 mg/kg and SC route of administration, suggest that doxycycline administered every 24 h could be effective against bacterial infections with MICs of 0.125 and 0.5 µg/mL. However, multi-dose and pharmacodynamic studies are needed to further evaluate the efficacy of using doxycycline in alpacas.

## 1. Introduction

The alpaca (Vicugna pacos), a camelid native to the Americas, has garnered significant interest in veterinary medicine due to its economic importance in South America, particularly for its highly valuable fiber production [1,2], as well as other economically significant products such as leather, fur, milk, manure and meat [3,4]. Alpaca fiber is highly appreciated in the global textile industry, with clothing and items made from it considered luxury goods [5]. This underlines its status as an important socio-economic resource in Latin American countries, where poverty rates among those involved in its exploitation are particularly high [6]. Peru has the largest population of alpacas and is the world’s leading exporter of their fiber [7,8,9]. However, alpaca breeding has expanded globally, with significant populations in Australia, Canada, the United States, New Zealand, the United Kingdom, and several European countries, including Germany, France, the Netherlands, and Sweden. Their growing popularity as companion, exhibition, and leisure animals has contributed to this expansion [2,10,11,12]. Despite their economic importance and growing numbers, alpacas are currently considered a minor species compared to other farm animals, resulting in a lack of research, development and marketing of medicines for this species.

Respiratory and gastrointestinal infections are the leading causes of morbidity and mortality in alpacas, particularly impacting juveniles more than adults [11]. Among the most prominent infections in young and neonatal animals, enterotoxaemia and acute respiratory conditions are of particular concern [13]. Several studies [14,15,16] have identified some of the agents responsible for these respiratory pathologies, including pathogenic bacteria such as *Streptococcus pneumonia*, *Pasteurella multocida* and *Mannheimia haemolytica*, while other researchers have highlighted *Clostridium perfringens* types A and C and *E. coli* [17,18,19]. These infections present a major health challenge for young alpacas, leading to significant economic losses [13]. The selection of antimicrobial treatments for alpacas is generally based on an empirical approach, aiming to provide broad-spectrum coverage to ensure clinical success. This approach is primarily driven by the paucity of pharmacokinetic studies available for this species, requiring the extrapolation of doses from studies conducted in other domestic species. However, this practice may entail several risks, including potential adverse effects, due to the different physiological characteristics of alpacas compared to other domestic animals [20]. Furthermore, the inappropriate use of these drugs has led to a serious problem, known as antimicrobial resistance, as defined by the World Health Organization (WHO), which has become a significant threat to public health [21,22]. Therefore, the development of species-specific pharmacokinetic studies is essential to achieve the rational and appropriate use of antimicrobials, which will help to curb the spread of this phenomenon [23].

In this regard, the European Medicines Agency’s Ad Hoc Expert Group on Antimicrobial Guidance (AMEG) has classified antibiotics based on their impact on public health through the development of antimicrobial resistance when used in animals and the necessity for their veterinary use. The AMEG categorization includes four categories: A (Avoid), B (Restrict), C (Caution) and D (Prudence). The risk of increasing antimicrobial resistance decreases from category A to category D. Category D, which includes tetracyclines such as doxycycline, should be used as a first-choice treatment if possible, and only when medically necessary [24]. This antibiotic belongs to the tetracycline group, which is one of the most widely used groups of antimicrobials in veterinary practice due to its promising pharmacokinetic characteristics and broad spectrum of activity against a variety of Gram-negative, Gram-positive and intracellular bacteria [25,26].

The kinetic dispositions of doxycycline have been investigated in many species of veterinary interest, including goats, sheep, rabbits, calves, horses, donkeys and pigs, and have shown adequate pharmacokinetic properties and an acceptable safety profile [27,28,29,30,31,32,33,34,35]. However, the pharmacokinetics of doxycycline have not been investigated in alpacas or other camelid species. Only two previous studies evaluated the pharmacokinetics of other tetracycline (oxytetracycline) in camels after IV and IM administration [36,37]. However, the tetracycline group is used in veterinary clinical practice for the treatment of infections in South American Camelids [38]. Therefore, doxycycline could be a useful antimicrobial for the treatment of infections caused by microorganisms sensitive to this antibiotic in alpacas. In view of the above facts, this study was designed to determine the pharmacokinetics of doxycycline in alpacas after intravenous and subcutaneous administration at doses of 5 and 20 mg/kg, respectively.

## 2. Results

### 2.1. Animals

All alpacas remained in optimal health and general condition throughout the experimental period, with no changes in behavior, activity or appetite detected by visual examination after IV and SC administration of the drug. There were no indications of discomfort or inflammation at the injection sites after both IV and SC administrations. The assessment included measuring vein swelling, monitoring changes in skin temperature and evaluating for pain at the injection sites.

### 2.2. Analytical Method

The retention times for doxycycline and tetracycline were measured at 2.1 min and 1.2 min, respectively. Calibration curves were plotted using the area ratio of doxycycline to tetracycline against the nominal concentration of doxycycline in µg/mL. The concentration range demonstrated a linear relationship from 0.1 to 100 µg/mL. The linear regression equation of doxycycline obtained a regression coefficient value of R^2^ = 0.996. The lower limit of quantification (LLOQ) was established at 0.1 μg/mL, while the limit of detection (LOD) was determined to be 0.035 μg/mL. The results are presented in Table 1. Based on the above, the proposed method proves to be suitable and sufficiently sensitive for quantifying doxycycline concentrations in plasma using HPLC with UV detection.

### 2.3. Pharmacokinetics

**Figure 1:**
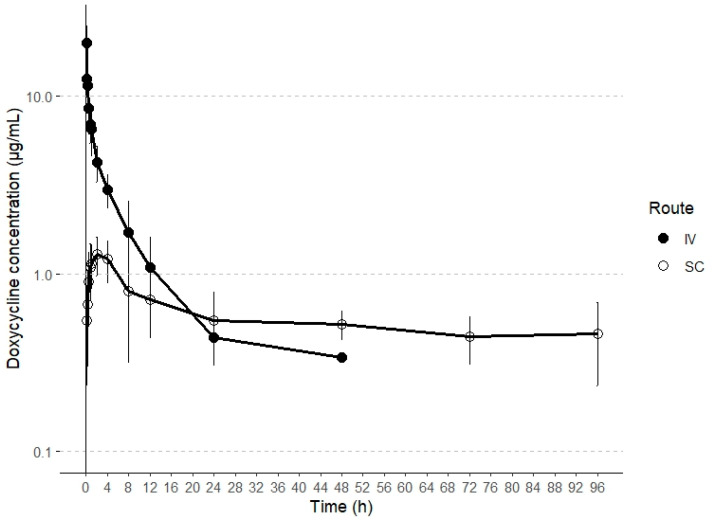
Semilogarithmic plots of intravenous and subcutaneous concentrations (mean ± SD) of doxycycline in alpacas at a single dose of 5 and 20 mg/kg, respectively (n = 6).

This report represents the first characterization of the disposition kinetics of doxycycline following both intravenous (IV) and subcutaneous (SC) administration in alpacas. Plasma concentrations exceeding the lower limit of quantification (LLOQ) were detected until the collection time points of 24 h and 96 h post-administration for IV and SC routes, respectively. The plasma concentration profiles of doxycycline following both IV and SC administrations are illustrated in Figure 1.

Mean (±SD) non-compartmental pharmacokinetic parameters are presented in Table 2.

## 3. Discussion

Compared to other farm animals, few drugs are approved for use in alpacas. The antimicrobial drug doses for this species are typically based on elimination characteristics determined in sheep and goats, due to their similar weight and nutritional requirements. However, it is worth noting that the Tylopoda (camelid family) and the Bovidae diverged phylogenetically more than 55 million years ago [39].

Many antimicrobials administered to alpacas are considered off-label, with dosing regimens, withdrawal times and indications often derived from data established in other species. The established doses and administration regimens are generally appropriate, but they are not always accurately extrapolated directly from other ruminants. For example, pharmacokinetic studies with other antimicrobials, such as enrofloxacin or ceftiofur [40,41,42], have shown considerable variation in the half-lives and distribution volumes of both drugs when compared to cattle and sheep. When drug doses are extrapolated between species, it is assumed that pharmacokinetic parameters remain consistent, which may not be accurate. Alpacas exhibit distinct anatomical and physiological differences that lead to variations in the pharmacokinetics compared to other species [41].

Further research is still needed on antimicrobial dosing in alpacas to establish more effective therapeutic regimens. Therefore, to optimize doxycycline dosing, it is essential to measure the concentration of this antibiotic in different biological fluids. Currently, there is no available information on the pharmacokinetics of doxycycline specifically in alpacas.

After IV administration, the obtained V_z_ value was 0.90 L/kg, suggesting a good distribution of this antibiotic in alpacas. Slightly lower values were obtained with the same formulation in goat (V_z_ = 0.85 L/kg) and sheep (V_z_ = 0.84 L/kg) [27,28]. However, higher values were obtained in ruminants (V_z_ in sheep: 1.76 L/Kg [34]; V_z_ in calves: 1.31 L/Kg [43]) and in camels (Vd = 1.41 L/Kg [37]) with other salts of doxycycline (not hyclate). The better distribution of doxycycline hyclate in this study compared to other ruminants and camelids may be attributed to a possible lower binding affinity to plasma proteins. The Cl obtained after IV administration was 0.11 L/h/Kg, which is lower than values recorded in small ruminants (Cl in sheep: 0.28 L/h/Kg [28]; Cl in goats: 0.19 L/h/Kg [27]), suggesting a lower metabolic rate in alpacas. The glomerular filtration rate (GFR) in alpacas is approximately 1 mL/kg/min, comparable to camels but notably lower than in goats (4 mL/kg/min). This suggests that drugs eliminated via the kidneys may be more prone to accumulation in alpacas compared to other ruminants [41].

Following IV administration of doxycycline, the half-life obtained was 5.06 h, which is higher than the values reported in previous studies with ruminants using the same doxycycline formulation, where the t_½_ was 2.14 h in goats [27] and 2.81 h in sheep [28]. Other studies in goats and camels report similar values to those observed in this study, with half-lives of 4.11 h [44], 4.39 h [29], 7.7 h [36] and 4.62 h for doxycycline hydrochloride [45].

The half-life obtained following IV administration was significantly different from that observed following SC administration (41.97 h). After SC administration, the drug’s half-life was longer compared to intravenous administration, likely due to a longer absorption phase. These findings align with previous studies in goats (40.92 h) [44], cows (40.81 h) [31], sheep (41.02 h) [28] and camels [36].

The mean residence time (MRT) values exhibited a similar pattern. The MRT for IV administration (7.38 h) was higher than those reported in other studies with small ruminants [27,28,29,45]. For the SC route, the MRT (64.62 h) was slightly longer than those observed previously in goats (44.83 h) and cows (54.30 h) [31,44]. MRT values for extravascular administration were significantly longer than those observed after IV administration, indicating that the molecules’ transit time is extended due to the absorption process. This observation suggests that doxycycline may follow a flip-flop pharmacokinetic model, where absorption serves as the rate-limiting step for plasma clearance [46]. Furthermore, another study with the same doxycycline formulation in sheep [28] and using the same administration routes produced similar results, supporting the existence of a flip-flop pharmacokinetic model.

The maximum concentration (C_max_) after SC administration was 1.40 µg/mL and the time in which this maximum plasma concentration was reached (t_max_) was 1.92 h. When these values were compared with those obtained in sheep with the same formulation (C_max_ = 1.81 µg/mL and t_max_ = 2.80 h [28]), it was observed that doxycycline was absorbed more rapidly in alpacas, but to a lesser extent. This finding is further supported by the low bioavailability of doxycycline after SC injection, which was 36.89%. Low bioavailability of this antibiotic has also been described after oral administration in goats (31.39%; [29]) and sheep (35.77%; [34]), after IM administration in goats and sheep (45.60% and 31.00%, respectively [27,28]) and after SC administration in sheep (53.66%; [28]). As indicated above, our research group obtained low bioavailability values in small ruminants after parenteral administration of doxycycline. In these studies, doxycycline was an irritant after IM administration, causing discomfort at the injection site, which may have contributed to reduced absorption. Pain and irritation were also observed in another study after IM administration in goats [29].

**Figure 2:**
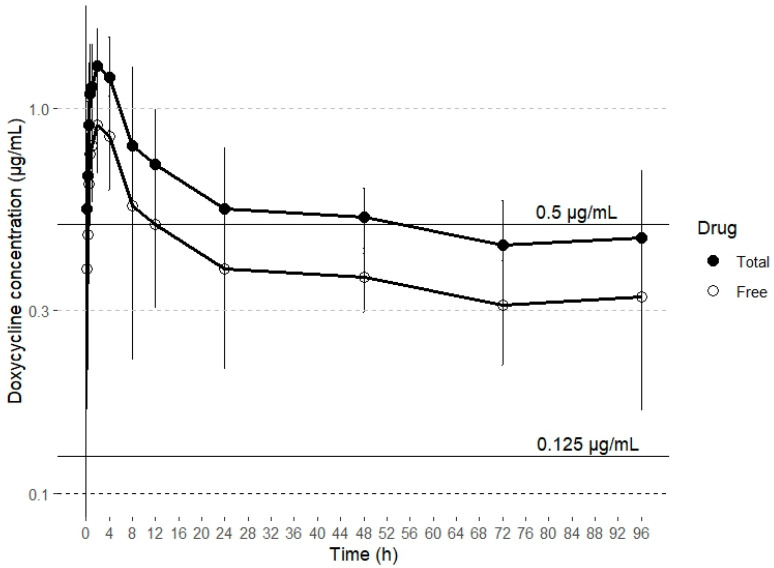
Concentration (mean ± SD) vs. time curve over 24 h for total drug doxycycline concentrations and estimated free drug doxycycline concentrations after subcutaneous administration of a single dose of 20 mg/kg. The lines represent target MICs of *C. pseudotuberculosis* (≤0.125 μg/mL) and *R. equi* (0.5 μg/mL).

Drugs can be categorized based on their antibacterial action into two groups: time-dependent and concentration-dependent agents. For concentration-dependent drugs, increasing the antibiotic concentration results in more rapid bacteria killing. In contrast, time-dependent drugs maintain a consistent kill rate, regardless of any increase in drug concentration [47]. Tetracyclines like doxycycline are traditionally regarded as time-dependent in their pharmacodynamics. Their efficacy is primarily determined by the duration for which the drug concentration at the site of action remains above the minimum inhibitory concentration (MIC), commonly referred to as T > MIC [34]. However, other publications suggest a concentration-dependent effect on bacterial growth, with the AUC _0–24_ /MIC surrogate marker correlating best with clinical efficacy [48,49]. In a study investigating doxycycline’s effects on *M. gallisepticum*, the drug demonstrated time-dependent behavior [50]. In contrast, a separate study on *M. hyopneumoniae* found that its action was better described as concentration dependent [49]. These findings suggest that doxycycline’s antibacterial activity against Gram-positive and Gram-negative pathogens may vary. At lower concentrations, the drug exhibits time-dependent killing, while at higher concentrations, it shows concentration-dependent kinetics [49]. Given these findings, doxycycline’s effectiveness is primarily linked to maintaining plasma concentrations that surpass the MIC by 1–5 times for 40–100% of the dosing interval, as well as achieving an AUC_0–24_ /MIC ratio of ≥ 125 [51]. However, AUC _0–24_ /MIC ratios lower than 125 have been necessary with this antimicrobial to achieve a bacteriostatic (59) and bactericidal (98) effect against *Haemophilus parasuis* in pigs [52]. It is important to note that only free doxycycline has antibacterial activity and that the study reports total plasma concentrations of doxycycline; therefore, all PK/PD ratios should be expressed in terms of free plasma concentration [53,54]. Consequently, this aspect should be considered when estimating PK/PD ratios. In goats, the binding ratio of doxycycline to plasma proteins is 33%, while in several other animal species, including sheep and calves, it is 90% or higher [26,34]. To date, there is no data on the plasma protein binding rate of doxycycline in alpacas. Some minimum inhibitory concentration (MIC) values for doxycycline against different bacterial strains isolated from alpacas have been reported in the literature. Specifically, in the case of a strain of *Rhodococcus equi* that caused a fatal infection in an adult alpaca, the MIC was 0.5 μg/mL [55]. For *Corynebacterium pseudotuberculosis,* the MIC obtained was ≤ 0.125 μg/mL [56]. Therefore, based on the plasma concentrations of doxycycline obtained after SC administration, and assuming a plasma protein binding similar to that reported in goats (~30%), doxycycline would maintain concentrations higher than the MICs reported for *C. pseudotuberculosis* (≤0.125 μg/mL) and *R. equi* (0.5 μg/mL) during 50% of the dosing interval, which has been established as 24 h (Figure 2).

## 4. Materials and Methods

### 4.1. Animals

Six healthy adult huacaya alpacas, three males and three females, from the Veterinary Teaching Farm of the Escuela Superior Politécnica del Chimborazo (Riobamba, Ecuador), weighing 60.5 ± 28.5 kg and aged 3–8 years, were selected for the study. All animals underwent a physical examination and body condition assessment prior to the start of the study, and health parameters were subsequently assessed at various time points (1, 10, 24, 48 and 72 h) after doxycycline injection. The alpacas were fed a drug-free diet for 21 days prior to the start of the study and were not given any treatment during the study period. One day prior to the study, all animals were shaved at the jugular vein for blood collection after IV or SC administration of the drug. In addition, animals assigned to the SC group were shaved on the back. Finally, the head, neck and base of the tail were marked with colored markers to distinguish the route of drug administration and the blood collection sequence. The bioethics committee of our institution approved the research protocol (CEEA 758/2021).

### 4.2. Experimental Design

A 2 × 2 crossover study was designed in two periods, with at least 21 days of washout between each period. First, male alpacas underwent a single subcutaneous (SC) administration of doxycycline, which was administered at a dose of 20 mg/kg of body weight. Specifically, the drug used was DFV Doxivet Injectable, a commercial product supplied by DIVASA-FARMAVIC (Barcelona, Spain). Female alpacas received a single intravenous (IV) administration of doxycycline at a dose of 5 mg/kg body weight. The formulation used was Vibravenosa, a 100 mg solution for injection produced by HOSPIRA INVICTA (Madrid, Spain). For the intravenous (IV) administration, the doxycycline was slowly administered as a bolus into the left jugular vein over a duration of approximately 1 min, and SC injections were administered under the skin of the back. After the wash-out period, the route of administration was switched, selecting the SC route for the females and the IV route for the males. The intramuscular (IM) route of administration was not evaluated in this study because of muscle irritation and lameness in previous studies in small ruminants using the same formulation [27,28]. Furthermore, in alpacas, the maximum volume to be administered intramuscularly is 3–5 mL; therefore, taking into account the weight of the animals used and the formulation used, it would have been necessary to use 2 to 3 injection sites, which could be impractical and potentially stressful for the animals.

Blood samples were collected from the right jugular vein into heparinized containers at 0 (pre-treatment), 0.083 (only after IV administration), 0.167, 0.25, 0.5, 0.75, 1, 1.5, 2, 4, 6, 8, 12, 24, 48, 72 and 96 h after administration. The tubes were carefully mixed to avoid hemolysis and centrifuged at 3500 rpm for 15 min. Plasma was deposited in 1.5 mL Eppendorf tubes, in duplicate, and stored at −40 °C until analysis. To assess damage at the site of administration according to the chosen route, the presence or absence of possible changes in skin temperature on the back or phlebitis in the jugular vein, and pain on palpation at both sites were checked.

### 4.3. Analytical Methods

Plasma doxycycline concentrations were quantified by high-performance liquid chromatography (HPLC) with ultraviolet detection, using a modified analytical method published by our research group [57]. Doxycycline and tetracycline hydrochloride (internal standard (IS)) were bought from Cymit Química (Barcelona, Spain) and were used for both quality control and calibration.

First, a total of 10 μL of internal standard (IS) solution, at a concentration of 10 μg/mL, was added to 200 μL of plasma. To precipitate proteins, a combination of 100 μL of a 1:2 solution of trifluoroacetic acid and methanol, as well as 100 μL of methanol, was added. The sample was vortexed for 20 s. This vigorous mixing ensures that the methanol and TFA solution is well-distributed throughout the plasma, facilitating protein precipitation and analyte extraction. Following the vortexing step, the sample was then sonicated for 6 min. After this, it was centrifuged for 10 min at 11,000 rpm. The clear supernatant was injected (50 μL per sample) into the HPLC system. The analysis was performed on a Thermo Scientific Ultimate 3000 HPLC system (Thermo Fisher Scientific, Waltham, MA, USA), featuring a quaternary gradient pump, variable wavelength detector, thermostatted column compartment, and an autosampler/autoinjector. Data acquisition and processing were conducted using Thermo Scientific Dionex Chromeleon software, version 7.2.10. A Restek Roc C18 column (5 µm, 150 × 4.6 mm; Restek, Bellefonte, PA, USA) was employed for the analysis. The HPLC method employed isocratic elution with a mobile phase composed of HPLC water and methanol (70:30), acidified to pH 2.0 with trifluoracetic acid (1 mL). The flow rate was maintained at 1.0 mL/min, the column temperature was set at 40 °C and detection was performed at 350 nm.

### 4.4. Method Validation

The validation of the method has been carried out on the basis of the FDA Guidance for Bio-analytical Method Validation [58]. The parameters assessed were as follows: precision, linearity, accuracy, limit of quantification (LOQ), limit of detection (LOD), selectivity and recovery. A previous publication from our group provides a complete description of the protocols used to validate each of these parameters and the acceptable coefficients of variation [54]. In brief, eight plasma concentrations of doxycycline and IS were analyzed to assess the linearity of the proposed method, with each level being analyzed in triplicate. Doxycycline’s lower limit of detection was determined based on the concentration yielding a signal-to-noise ratio of ≥3. The limit of quantitation was determined as the lowest concentration on the calibration curve with an accuracy of less than 20% CV. The precision and accuracy of the method were calculated by analyzing five replicates from four quality controls spiked with IS (intraday: five replicates of each concentration analyzed on the same day; interday: five replicates of each concentration analyzed across three consecutive days). Recovery tests were conducted by analyzing three concentrations, with five samples analyzed at each concentration level. Six drug-free plasma samples were analyzed to evaluate the selectivity of the method.

### 4.5. Pharmacokinetic Analysis

Any data below the LOQ were discarded. The calculation of noncompartmental parameters was performed using WinNonlin^TM^ (WinNonlin; Pharsight Corporation; Mountain View, CA, USA). The bioavailability (F) for the SC route was calculated using the following equation:

F (%) = (AUC_subcutaneous_/AUC_intravenous_) × (Dose_intravenous_/Dose_subcutaneous_) × 100.

The abbreviations and descriptions of each pharmacokinetic parameter can be found in the footnote of Table 2.

### 4.6. Statistical Analysis

The statistical analysis was conducted using Jamovi software, version 2.3.28. All pharmacokinetic data, with the exception of half-lives (which were determined using the harmonic mean), were expressed as arithmetic means and standard deviations. The Shapiro–Wilk test was performed to evaluate the normality of the data. A paired *t*-test was utilized to examine statistical differences between datasets when the data followed a normal distribution. In contrast, a Wilcoxon signed-rank sum test was employed for datasets that did not adhere to normality. Statistical significance was accepted at a *p*-value of less than 0.05.

## 5. Conclusions

This study showed no adverse effects associated with the administration of a single dose of doxycycline following IV (5 mg/kg) or SC (20 mg/kg) administration in alpacas. After SC injection, pharmacokinetic analysis showed a prolonged half-life, low clearance and good volume of distribution. Based on the published MICs for bacterial pathogens isolated from alpacas and the plasma concentrations of doxycycline obtained after SC administration, doxycycline could be useful for the treatment of infections caused by microorganisms susceptible to this antimicrobial agent. Despite these results, future studies are needed to establish repeated dosing regimens and clinical efficacy.

## Figures and Tables

**Table 1:** Validation parameters of doxycycline in plasma of alpacas.

Validation Parameters	Tildipirosin
Linearity range	0.1–100 µg/mL
Correlation coefficient, R^2^	0.996
Repeatability (CV)	<7.2%
Reproducibility (CV)	<6.9%
LLOD	0.035 µg/mL
LLOQ	0.1 µg/mL
Recovery	82.13%

**Table 2:** Pharmacokinetic parameters (mean ± SD) of doxycycline in six alpacas after a single dose of 5 mg/kg administered intravenously and 20 mg/kg subcutaneously.

Parameters (Units)	Intravenous			Subcutaneous		
C_0_ (µg/mL)	33.22	±	14.35			
λ_z_ (h^−1^)	0.137	±	0.084	0.017	±	0.004 ^a^
t_½λz_ (h) *	5.06			41.97 ^a^		
V_z_ (L/kg)	0.90	±	0.38			
V_ss_ (L/kg)	0.76	±	0.33			
Cl (L/h/kg)	0.11	±	0.03			
AUC_0–∞_ (µg·h/mL)	50.15	±	13.53	66.43	±	14.87 ^a^
MRT (h)	7.38	±	2.47	64.62	±	11.81 ^a^
MAT (h)				57.24	±	13.98
C_max_ (µg/mL)				1.40	±	0.30
t_max_ (h)				1.92	±	1.20
F (%)				36.83	±	17.64

^a^ There are significant differences with the IV administration (*p* < 0.05). * Harmonic mean. C_0_: concentration of the drug in plasma immediately after intravenous administration; λ_z_: the slowest elimination rate constant; t_½λz_: half-life associated with the terminal slope (λ_z_) of a semilogarithmic concentration versus time curve; V_z_: apparent volume of distribution calculated according to the method of the area; V_ss_: apparent volume of distribution in the steady state; Cl: total body clearance of the drug from plasma; AUC_0–∞_: the area under the plasma concentration versus time curve from zero to infinity; MRT: the mean residence time; MAT: the mean absorption time; C_max_: the peak or maximum plasma concentration after extravascular administration of the drug; t_max_: the time after extravascular administration to peak or maximum plasma concentration; F: the proportion of the administered dose that is available systemically (bioavailability). Significant differences were found between IV and SC administration in λz, AUC and MRT.

## Data Availability

Data are contained within the article.
